# Transcriptome‐wide RNA m6A methylation profiles in an endemic osteoarthropathy, Kashin‐Beck disease

**DOI:** 10.1111/jcmm.70047

**Published:** 2024-10-20

**Authors:** Qian Zhang, Xiaodong Yang, Xingxing Deng, Hui Niu, Yijun Zhao, Jinfeng Wen, Sen Wang, Huan Liu, Xiong Guo, Cuiyan Wu

**Affiliations:** ^1^ School of Public Health, Health Science Center Key Laboratory of Environmental and Endemic Diseases of National Health Commission of the People's Republic of China, Xi'an Jiaotong University Xi'an People's Republic of China; ^2^ Shaanxi Provincial Institute for Endemic Disease Prevention and Control Xi'an People's Republic of China

**Keywords:** differences in gene expression, Kashin‐Beck disease (KBD), N6‐methyladenosine (m6A), RNA‐seq, transcriptome

## Abstract

Kashin‐Beck disease (KBD) is a chronic degenerative, disabling disease of the bones and joints and its exact aetiology and pathogenesis remain uncertain. This study is to investigate the role of m6A modification in the pathogenesis of KBD. Combined analysis of m6A MeRIP‐Seq and RNA‐Seq were used to analyse human peripheral blood samples from three KBD patients and three normal controls (NC). Bioinformatic methods were used to analyse m6A‐modified differential genes and RT‐qPCR was performed to validate the mRNA expression of several KBD‐related genes. The results indicated that the total of 16,811 genes were modified by m6A in KBD group, of which 4882 genes were differential genes. A large number of differential genes were associated with regulation of transcription, signal transduction and protein binding. KEGG analysis showed that m6A‐enriched genes participated the pathways of Vitamin B6 metabolism, endocytosis and Rap 1 signalling pathway. There was a positive association between m6A abundance and levels of gene expression, that there were 6 hypermethylated and upregulated genes (hyper‐up), 23 hypomethylated and downregulated genes (hypo‐down) in KBD group compared with NC. In addition, the mRNA expression of levels of MMP8, IL32 and GPX1 were verified and the protein–protein interaction networks of these key factors were constructed. Our study showed that m6A modifications may play a vital role in modulating gene expression, which represents a new clue to reveal the pathogenesis of KBD.

## 1 INTRODUCTION

Kashin‐Beck disease (KBD) is an endemic chronic osteochondrosis. The clinical manifestations of KBD patients are polymorphic, symmetrical joint involvement. Patients are often accompanied by joint pain, thickening, deformation and muscle atrophy in the limbs.^1^ Severe patients even have deformities and short stature.^2^ The main pathological changes of KBD are banded or sheet necrosis in the epiphyseal growth plate cartilage and deep articular cartilage of multiple bones.^3^ The disease damages not only cartilage but also heart, skeletal muscle, bone marrow, blood vessel walls, stomach, endocrine glands and peripheral nerve.^4^ In past studies, researchers have identified more than 50 environmental risk factors for KBD. The biogeochemical theory, the theory of food mycotoxin poisoning, the theory of organic matter poisoning in drinking water and other etiological theories have been put forward,^5^, ^6^, ^7^, ^8^ but the pathogenesis of KBD is still not completely clear.

In recent years, epigenetic studies on KBD have gradually increased and it has been found that gene methylation plays an important role in the occurrence and development of the disease.^9^ A research found that m6A writer METTL3 was upregulated in endplate chondrocytes induced by IL‐1β, leading to dysfunctional cell vitality and metabolism, and accelerating the degeneration of endplate chondrocytes.^10^ Methylation writer WTAP regulated extracellular matrix (ECM) degradation, inflammation and antioxidation in human chondrocytes. In vitro dysregulated WTAP had positive effects on β‐catenin expression, which finally contributed to the cartilage injury in chondrocytes.^11^ A study revealed that the downregulation of m6A eraser ALKBH5 in osteoarthritic cartilage increased the m6A level of HS3ST3B1, ultimately inhibiting chondrocyte viability, promoting chondrocyte apoptosis.^12^ One report indicated that hypermethylation leads to downregulation of O6‐methylguanine‐DNA methyltransferase (MGMT) in chondrocytes from KBD patients. Meanwhile, gene silencing of MGMT results in DNA damage and apoptosis of chondrocytes.^13^ At present, and the research on DNA methylation is relatively in‐depth, meanwhile there is almost no research on RNA methylation of KBD. As the most common and most abundant type of RNA methylation modification, m6A modification plays an important role in cell homeostasis, physiological activities and many human diseases by regulating gene expression and other mechanisms.^14^, ^15^ Previous studies have shown that m6A modification plays a crucial role in osteochondral biology and the occurrence and development of osteochondral‐related diseases.^16^, ^17^ m6A modification in fibroblast‐like synoviocytes increased accompanying with the increase of METTL3 expression in OA. METTL3 is involved in OA by regulating the inflammatory response. Overexpression of METTL3 can increase the expression of metalloproteinase MMP1 and MMP3, and decrease the expression of MMP13, TIMP‐1 and TIMP‐2 at the mRNA and protein levels, thereby affecting the degradation of ECM in OA.^18^ A study found that samples tested from healthy and OA showed different expression patterns of m6A regulators. YTHDF3 expression was upregulated in OA samples, and 23 m6A regulators mediate three distinct patterns of RNA modification.^19^ KBD and OA are both bone and joint diseases, and have similar clinical and pathological manifestations.^20^ The above studies suggest that m6A may play an important role in the occurrence and development of KBD. However, the m6A methylation profile of KBD has still not be described.

In our study, we showed m6A transcriptome map and its potential functions and mechanisms in KBD through RNA high‐throughput sequencing (RNA‐seq) and methylated RNA immunoprecipitation sequencing (m6A MeRIP‐Seq) analysis. Our study provided more perspectives and clues of methylation regulation mechanism in the pathogenesis of KBD.

## 2 MATERIALS AND METHODS

### 2.1 Subject information and sample collection

Human peripheral blood samples were collected from three patients diagnosed with KBD and three normal controls (NC) who were all from the KBD‐affected areas. The diagnosis of KBD was based on the diagnostic criteria of KBD in China (WS/T 207–2010). The peripheral blood specimens (5 mL) were collected into an anticoagulation tube and immediately added with Trizol reagent (Invitrogen, CA, USA) and then stored at −80°C. Specific information of the subjects is in Table S1. All participants were aware of this study and provided written informed consent. This investigation was approved by the Human Ethics Committee of Xi'an Jiaotong University.

### 2.2 MeRIP‐seq and RNA‐seq

Total RNA was extracted from peripheral blood with Trizol reagent (Invitrogen, CA, USA), and its concentration and purity were determined by using NanoDrop ND‐1000 (NanoDrop, Wilmington, DE, USA). Epicentre Ribo‐Zero Gold Kit (Illumina, San Diego, USA) were used to deplete ribosomal RNA. After fragmented into small pieces using Magnesium RNA Fragmentation Module (NEB, cat. e6150, USA) under 86°C 7 min, RNAs were mixed with Dynabeads Antibody Coupling Kit (Thermo Fisher, CA, USA) and m6A antibody in IP buffer (50 mM Tris–HCl, 750 mM NaCl and 0.5% Igepal CA‐630). Then, the IP RNA was reverse‐transcribed to cDNA using SuperScript™ II Reverse Transcriptase (Invitrogen, cat. 1,896,649, USA). The PCR procedure was set as follows: pre‐denaturation at 95°C for 3 min, denaturation at 8 cycles of each 98°C for 15 s, annealing at 60°C for 15 s, extension at 72°C for 30 s and finally extension at 72°C for 5 min. Subsequently, double‐terminal sequencing were conducted used Illumina Novaseq™ 6000 according to standard operation with LC Bio Technology CO. Ltd. (Hangzhou, China).

### 2.3 Bioinformatics analysis process

**FIGURE 1:**
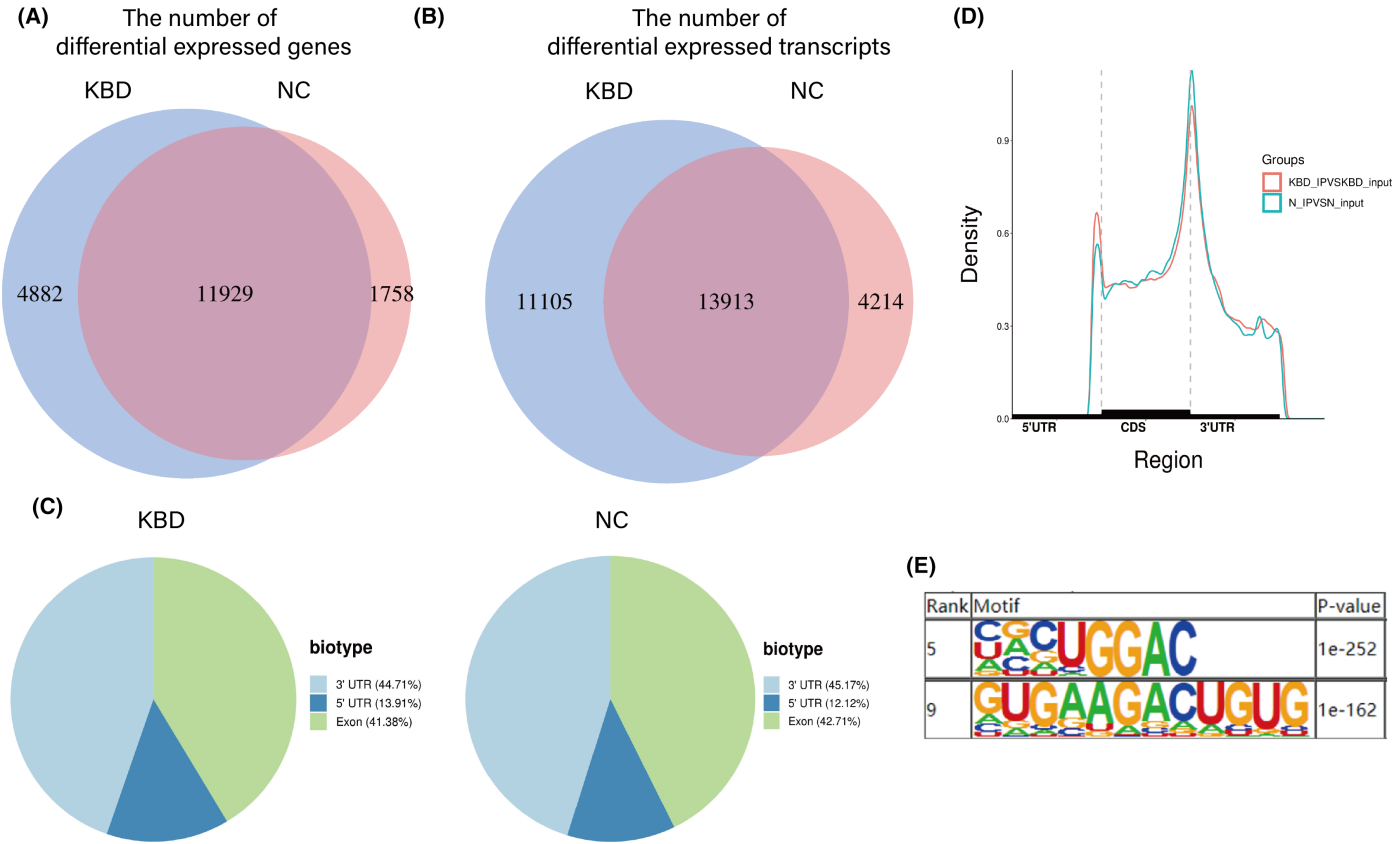
Overview of m6A modification of KBD patients and normal controls. (A, B) Venn diagrams showing overlaps of m6A‐modified genes and m6A‐modified transcripts. (C) Distribution of m6A peaks on different gene elements in KBD group and control group. (D) Difference in the density of m6A peaks along the gene between KBD group and control group. Each gene was divided into three parts: 5’‐UTR, CDS, and 3’‐UTR. (E) Sequencing logo displaying RRACH and GGAC conserved sequence motifs for the m6A peak regions, with *P* values of 1*e^−252^ and 1*e^−162^, respectively.

**FIGURE 2:**
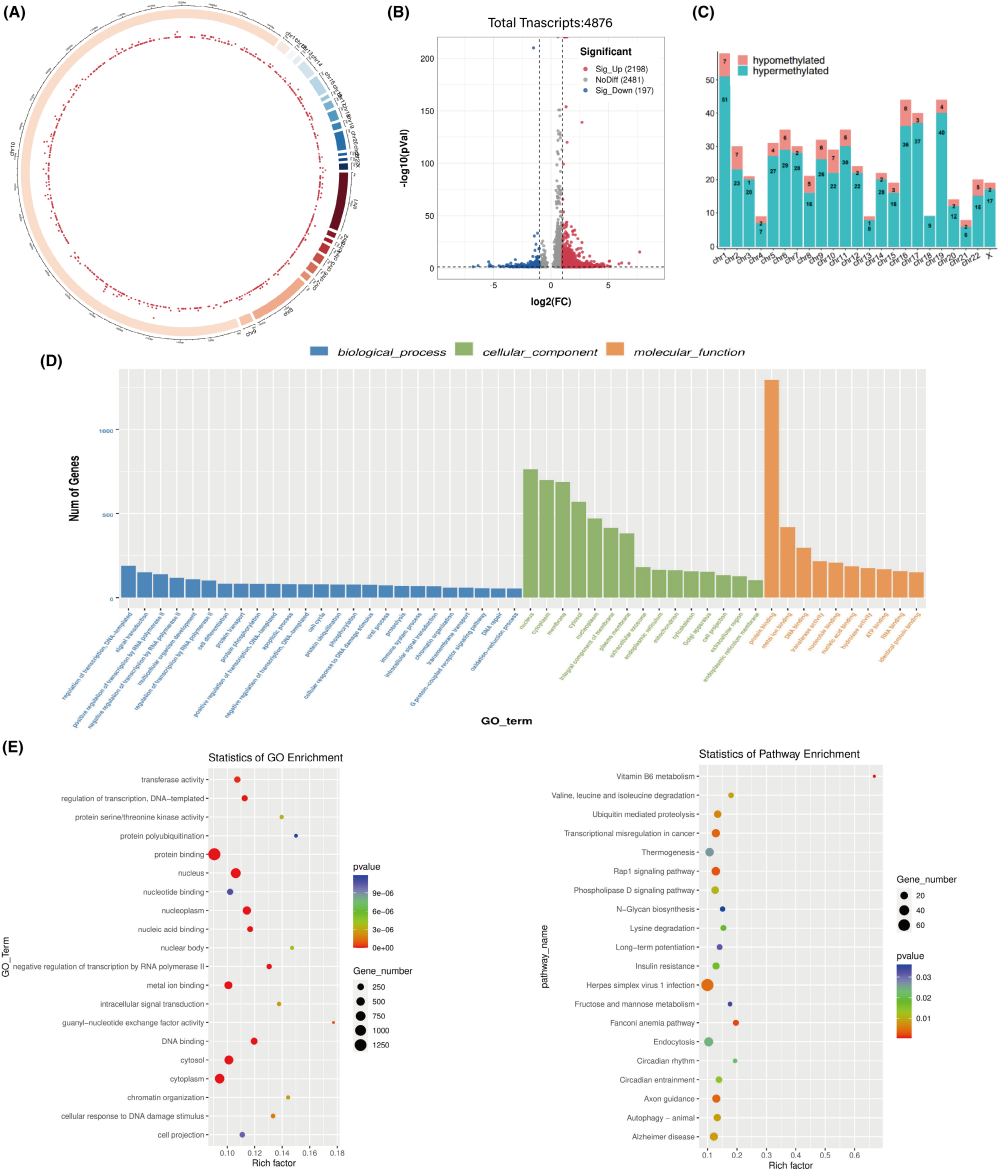
Distribution and topology pattern of m6A peaks along transcripts and chromatin of KBD patients and normal controls. (A) Circos plot showing the global distribution of m6A peaks, in human genome of KBD group and control group. (B) Volcano plots showing different m6A peaks between KBD group and normal group. (C) The distribution of hypermethylated and hypomethylated m6A‐modified peaks with significance along the chromosome of KBD. (D) Differential methylated peaks GO enrichment analysis, and the top 20 significant GO enrichment terms. (E) The top 20 significant KEGG enrichment pathways.

**FIGURE 3:**
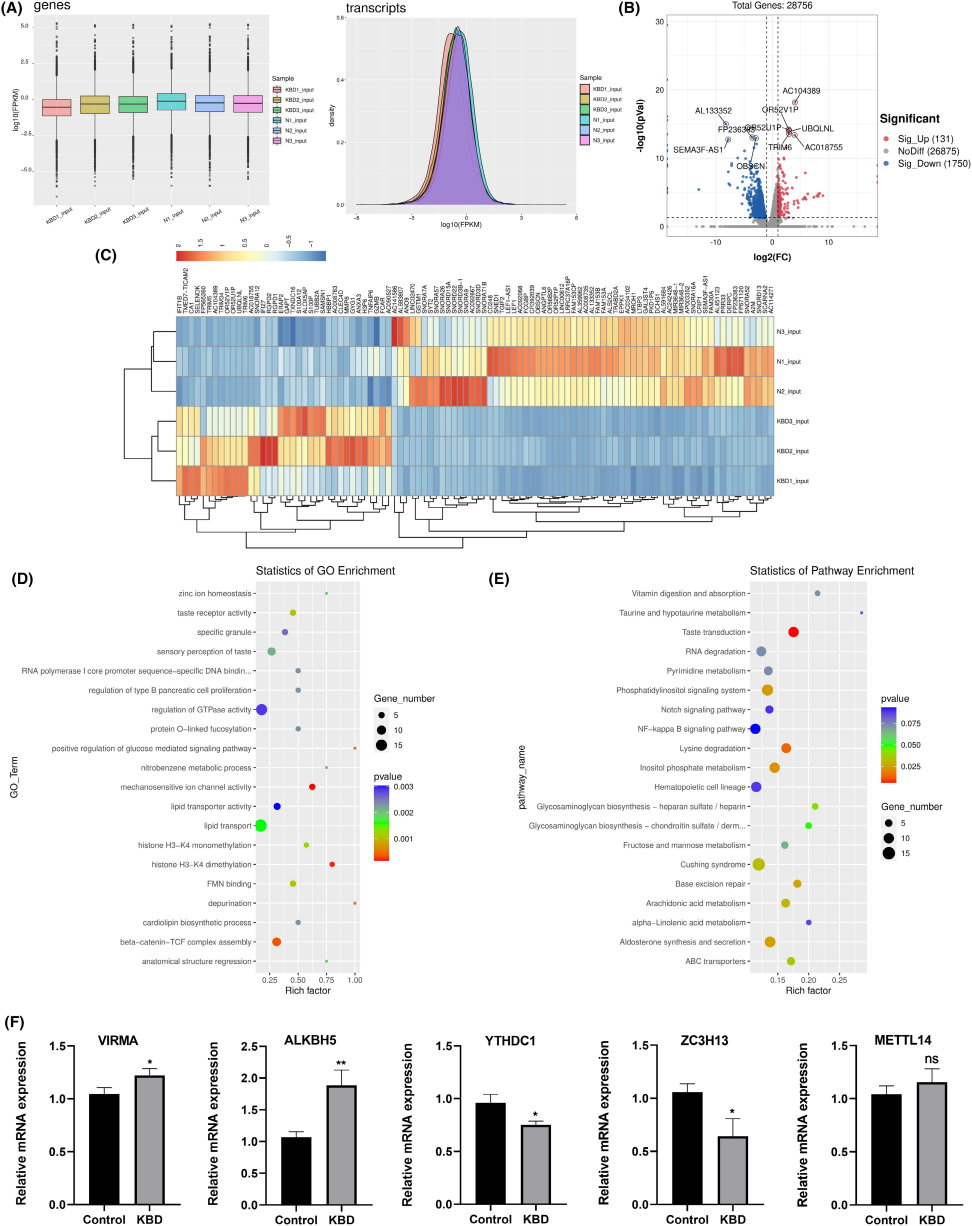
Genome‐wide differential analysis between KBD patients and normal controls. (A) Overall gene expression box plot and transcriptome expression density map of KBD group and control group. (B) Volcano plot of differentially expressed genes. (C) Differential gene cluster analysis heat map, the colour from blue to yellow tored indicates the expression level (log_10_(FPKM+1)) from low to high. (D) The top 20 significantly GO enrichment terms for significantly differentially expressed genes. (E) The top 20 enriched pathways of significantly differentially expressed genes. (F) mRNA expressions of VIRMA, ALKBH5, YTHDC1, ZC3H13 and METTL14 in KBD compared to normal control. The *P* values are 0.028, 0.0051, 0.0149, 0.0175, and 0.2532, respectively.

**FIGURE 4:**
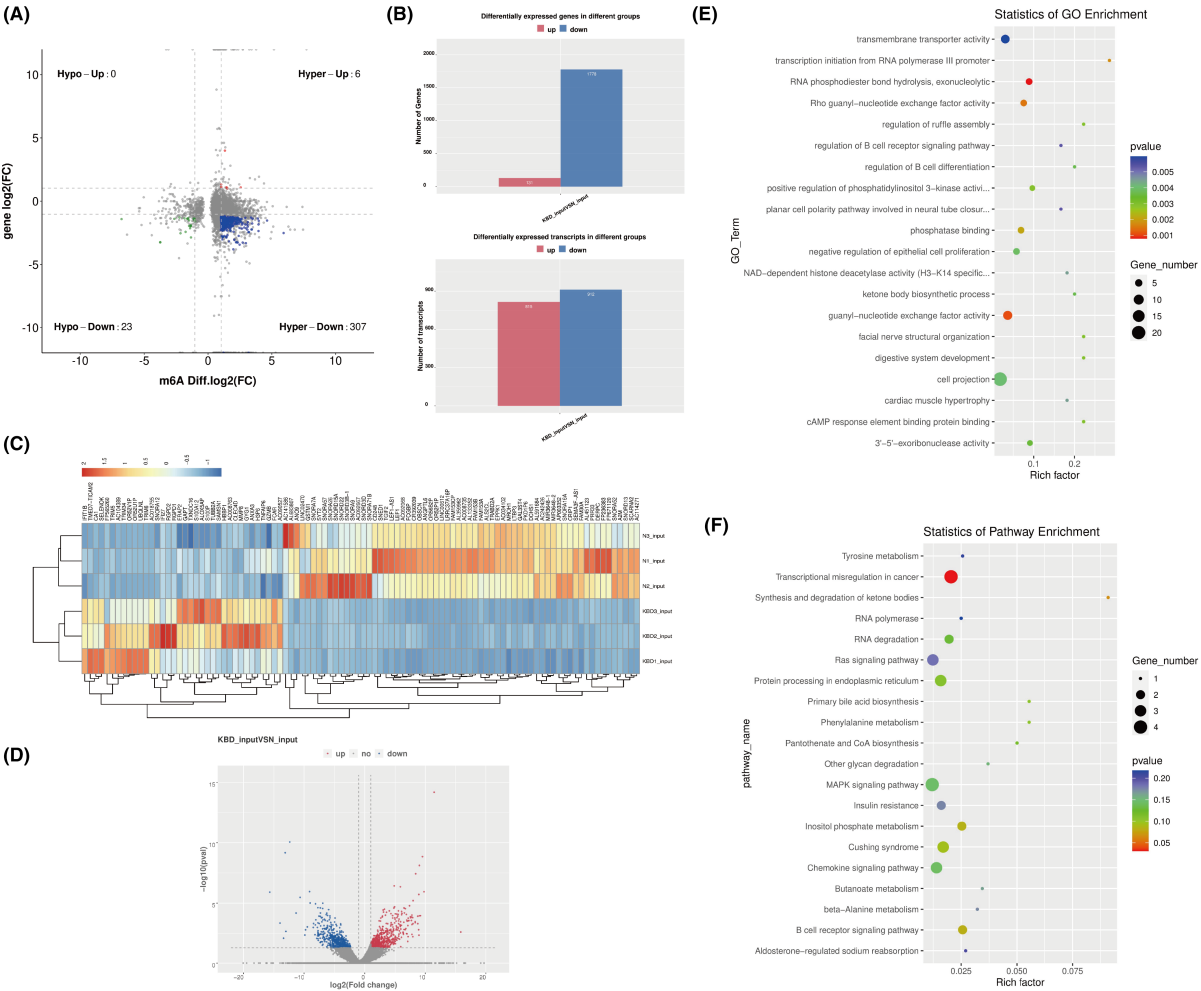
Conjoint analysis of m6A modification and gene expression. (A) Four‐quadrant diagram showed the differentially methylated genes and differentially expressed genes in KBD group and control group. (B) Bar plot of differentially expressed genes and transcripts in KBD group and control group. (C) Heat map under cluster analysis of differentially expressed gene. The colour from blue to yellow to red indicates the expression level (log_10_(FPKM+1)) from low to high. (D) Volcano of differentially expressed transcripts in KBD group and control group. (E) Major enrichment and meaningful GO terms of differentially methylated genes and differentially expressed genes in KBD. (F) The top 20 significant KEGG pathways in KBD.

The fastp (https://github.com/OpenGene/fastp) software was used to control the quality of the raw data. Then, the reads of each sample were mapped to the reference genome Homo sapiens (Version: v101) by using HISAT2 (http://daehwankimlab.github.io/hisat2). The analysis of peak calling was performed by R package exome Peak (https://bioconductor.org/packages/exomePeak) and the data visualization showed by IGV software (http://www.igv.org). The distribution of called peaks and adjacent genes were annotated by using R package ChIP seeker (https://bioconductor.org/packages/ChIPseeker). MEME (http://meme‐suite.org) and HOMER (http://homer.ucsd.edu/homer/motif) were applied for motif analysis. Then String Tie (https://ccb.jhu.edu/software/stringtie) was used to quantify the expression level of mRNAs. Taking∣log_2_FC∣ >2 and *p* <0.05 as the standard, R package edge R (https://bioconductor.org/packages/edgeR) was used for differentially expressed mRNAs analysis. All screening conditions in our subsequent studies were∣log_2_FC∣ > 2 and *p* < 0.05. The graphics drawing tools in Figures 1, 2, 3, 4 are all using the OmicStudio tools at https://www.omicstudio.cn/tool/.

### 2.4 Gene ontology (GO) and Kyoto Encyclopedia of Genes and Genomes (KEGG) analyses

GO and KEGG path analysis of m6A peaks and mRNAs. GO analyzes including molecular functions, biological processes and cellular components were performed. *p* < 0.05 indicates the significance of GO term enrichment. Based on the KEGG database, a pathway analysis was performed to analyse potential functions. GO and pathway enrichment analysis was also performed based on differentially regulated m6A peaks and mRNAs. GO seq R package was performed on the Gene Ontology (GO, http://www.geneontology.org/) enrichment analysis for the differentially expressed genes (DEGs) (Young et al., 2010). The KEGG database (http://www.genome.jp/kegg/) is a major resource for learning high‐level functions and utilities of biological systems. The statistical enrichment tests for genes of differential expression in the KEGG pathways were used in the KOBAS software.

### 2.5 Conjoint analyses

The upregulated and downregulated genes in RNA‐seq combined with their changes in m6A peak abundance. The data were integrated to count genes that had both changes in the level of m6A methylation modification and changes in the level of gene expression. Genes that had a multiplicity of difference between groups, were both more than two fold and bothc had *p*‐values less than 0.05. The prescribed conditions of |different peak. log_2_(FC)| ≥ 1, different peak. *P* value <0.05 and |different expression gene. log_2_(FC)| ≥ 1, different expression gene. *P* value <0.05 were considered to have differentiation in both methylation levels and transcriptional levels between KBD group and normal group.

### 2.6 Quantitative real‐time PCR

Total RNA was treated with gDNA Clean Reagent (code no. AG11705; Accurate). First‐strand cDNA was synthesized using Evo M‐MLV RT Kit (code no. AG11705; Accurate) according to the manufacturer's instructions. The RNA purity and concentration were measured with a Nano Drop 2000 (Thermo Fisher Scientific). RT‐qPCR was performed in three biological replicates three technical replicates using CFX96Real‐Time PCR Detection System (Bio‐Rad) with SYBR® Green Premix Pro Taq HS qPCR Kit (code no. AG11701; Accurate), the GAPDH was used as an internal control. The gene expression levels were estimated using the 2^−ΔΔCt^. The primers used for RT‐qPCR are listed in Table S3. The SPSS 22 software package was used to evaluate statistics. T test assessed the significance of the differences between all of the groups. Statistically significant was the degree of probability *p* < 0.05.

## 3 RESULTS

### 3.1 Characteristics of m6A‐MeRIP‐Seq transcriptome‐wide distributions in KBD patients and NC

At the gene level, 16,811 and 13,687 genes were modified by m6A in the KBD group and the control group and there were 11,929 overlapping genes. At the transcriptome level, 25,018 and 18,127 transcripts were modified by m6A in the KBD group and the control group, and there were 13,913 transcripts in the overlapping part (Figure 1A,B). Similar to the binding sites of METTL3/METTL14 complex in RNAs, the distribution of m6A peak mainly enriched in coding sequence (CDS) and 3′UTRs of mRNA in our study, while most of them preferred to locate in the region of stop codon and the beginning of 3′UTRs, which had no significant difference in two groups (Figure 1C,D). Besides, the differentially methylated m6A sites in KBD group most enriched in the 3′UTRs (44.71%), followed by the exon (41.38%). As is shown in the motif logo, the m6A‐related proteins were most likely to combine with the RRACH (R = A/G, H = A/C/U) and GGAC sequence of RNAs (Figure 1E).

### 3.2 Differential transcript m6A modification in KBD patients and NC

At the genome level, 603 m6A‐methylated genes were unevenly distributed across each chromosome (Figure 2A). In total, there were 2198 hypermethylated and 197 hypomethylated peaks of transcripts (Figure 2B). The top 20 differently methylated m6A peaks were showed in Table 1. Differentially expressed methylated m6A peaks are distributed on 23 chromosomes except Y chromosome, with the most on chromosome 1 and the least on chromosome 21. In the m6A peaks of all chromosomes, hypermethylated peaks are generally more than hypomethylated peaks (Figure 2C). Using GO enrichment to analyse biological process, cellular component and molecular function in which these differentially methylated peaks enriched, it found that a large number of those differential genes were associated with regulation of metal ion binding, DNA binding and transferase activity, while the greatest rich factor was related to the process of protein binding (Figure 2D). KEGG analysis were used to further research the biology function of those genes with differentially m6A modification. The results showed that m6A‐enriched genes participated the pathways of Vitamin B6 metabolism, Rap1 signalling pathway and Fanconi anaemia pathway (Figure 2E). Previous studies have shown that Wnt signalling pathway, TGF‐β signalling pathway, PI3K‐Akt signalling pathway, p53 signalling pathway and Apoptosis Autophagy are strongly correlated with the pathogenesis of KBD. Simultaneously screened genes with significantly altered expression in KBD related pathways were listed in Table 2.

**TABLE 1:** The top 20 m6A‐modified peaks for KBD patients compared to normal controls.

Gene ID	Gene name	Chr	Log_2_FC	*p* value	Regulation
ENSG00000286448	AP006222	Chr1	7.72	3.16E‐16	Hyper
ENSG00000228960	OR2A9P	Chr7	7.44	2.04E‐05	Hyper
ENSG00000198416	ZNF658B	Chr9	7.39	4.37E‐07	Hyper
ENSG00000274553	AC138035	Chr5	6.30	3.09E‐07	Hyper
ENSG00000181625	SLX1B	Chr16	5.92	8.51E‐05	Hyper
ENSG00000257545	AC079385	Chr12	5.30	1.86E‐06	Hyper
ENSG00000248610	HSPA8P4	Chr5	5.17	1.41E‐02	Hyper
ENSG00000168916	ZNF608	Chr5	5.07	2.19E‐07	Hyper
ENSG00000239577	RN7SL388P	X	4.98	1.29E‐02	Hyper
ENSG00000271797	AC008494	Chr5	4.91	3.72E‐03	Hyper
ENSG00000125551	PLGLB2	Chr2	−4.20	1.32E‐02	Hypo
ENSG00000197665	AC007952	Chr17	−4.39	3.24E‐02	Hypo
ENSG00000268593	AC008749	Chr19	−4.52	4.68E‐03	Hypo
ENSG00000243709	LEFTY1	Chr1	−5.04	2.69E‐02	Hypo
ENSG00000230317	LINC01284	X	−5.21	5.75E‐03	Hypo
ENSG00000238260	AL513320	Chr1	−5.33	3.47E‐04	Hypo
ENSG00000015568	RGPD5	Chr2	−5.39	1.58E‐02	Hypo
ENSG00000154874	CCDC144B	Chr17	−5.42	8.32E‐07	Hypo
ENSG00000260784	AC026150	Chr15	−6.34	3.16E‐03	Hypo
ENSG00000160223	ICOSLG	Chr21	−6.77	3.55E‐02	Hypo

Abbreviation: Chr, Chromosome.

**TABLE 2:** Significantly altered pathways associated with KBD patients compared to normal controls.

No.	Pathway function	Gene name	Gene ID	Chr	Log_2_FC	*p* value
1	Wnt signalling pathway	RNF43	ENSG00000108375	Chr17	2.65	3.63E‐04
		PRKACA	ENSG00000308677	Chr19	2.48	7.94E‐15
		VANGL1	ENST00000173218	Chr1	2.25	8.32E‐07
		CSNK2A1	ENST00000400227	Chr20	−2.51	2.04E‐02
2	TGF‐β signalling pathway	LEFTY1	ENSG00000243709	Chr1	−5.04	2.69E‐02
3	PI3K‐Akt signalling pathway	THBS3	ENSG00000169231	Chr1	2.68	4.27E‐04
		IRS1	ENSG00000169047	Chr2	2.54	7.76E‐04
		CREB3	ENSG00000107175	Chr9	2.49	3.80E‐03
		CD19	ENSG00000177455	Chr16	2.29	5.50E‐03
4	p53 signalling pathway	CCNG2	ENSG00000138764	Chr4	3.66	3.09E‐02
5	Apoptosis	DFFA	ENSG00000160049	Chr1	3.91	1.32E‐02
6	Autophagy	IRS1	ENSG00000169047	Chr2	2.54	7.76E‐04

	PRKACA	ENST00000308677	Chr19	2.48	7.94E‐15

Abbreviation: Chr, Chromosome.

### 3.3 The DEGs between KBD patients and NC

The results of whole‐genome sequencing showed that the expression of gene abundance between the KBD patients and NC were balanced and there was no significant difference (Figure 3A). The volcano plots showed the significantly different gene expression in the KBD group compared with the control group, including 131 up‐regulated genes and 1750 down‐regulated genes. Among these, the top three up‐regulated genes with significant differences were AC104389, OR52V1P and OR52U1P, while the top three down‐regulated genes were AL133352, FP236383, OBSCN (Figure 3B). Performing cluster analysis on these DEGs and using log_10_ (FPKM+1) to represent the gene expression level, the top 100 DEGs were identified in KBD group and the control group (Figure 3C). In addition, the DEGs were analysed by GO ontology and KEGG pathway (Figure 3D). The top 3 most significant functional annotations were mechanosensitive ion channel activity, histone H3‐K4 dimethylation, beta‐catenin‐TCF complex assembly. Meanwhile, pathway exploration revealed taste transduction, lysine degradation and other pathways related to the aetiology of KBD (Figure 3E). Five significantly changed m6A regulators of KBD in sequencing analysis (Table S2) were verified through qPCR. The results of qPCR were similar with the RNA‐seq. The expressions of VIRMA and ALKBH5 were significantly up‐regulated, while YTHDC1 and ZC3H13 were significantly down‐regulate in KBD compared to the NC. The expression of METTL14 was not significantly different between two groups (Figure 3F).

### 3.4 Conjoint analysis of m6A methylation and gene expression

To further explore the relationship between m6A modification and gene expression, we combined the results of MeRIP‐seq and RNA‐seq. As shown in the Four‐quadrant diagram, there were 6 hypermethylated and upregulated (hyper‐up) genes, 23 hypomethylated and downregulated (hypo‐down) genes, 307 hypermethylated and downregulated (hyper‐down) genes (Figure 4A), among which hypermethylated but downregulated genes accounts for a large part. There were 131 up‐regulated and 1778 down‐regulated DEGs. At the transcriptome level, there were 815 up‐regulated and 912 down‐regulated differentially expressed transcripts (Figure 4B). The top genes with differences in m6A regulation and gene expression in the combined analysis were listed in Table 3. No hypomethylated but upregulated genes meet the screening criteria and only four hypermethylated and upregulated genes meet the screening criteria. The top 50 up‐regulated and top 50 down‐regulated genes were analysed under clustering analysis (Figure 4C). It was shown that the expressions of IFIT1B, TMED7‐TICAM2 and CA1 were all decreased in the NC group, whereas their expressions were all increased in the KBD group. Differentially expressed transcripts were presented in the volcano plot (Figure 4D). The GO and KEGG pathway analysis for these m6A‐modifided and DEGs significantly involved  RNA phosphodiester bond hydrolysis, exonucleolytic, guanyl‐nucleotide exchange factor activity and so on (Figure 4E). Meanwhile, the top 20 biological enrichment of KEGG pathway analysis indicated that DEGs were substantially related to transcriptional misregulation in cancer, inositol phosphate metabolism and B cell receptor signalling pathway (Figure 4F).

**TABLE 3:** Genome‐wide differentially expressed genes and m6A modification levels in KBD patients compared with normal controls.

Gene ID	Gene name	m6A regulation	Gene regulation	Log_2_FC	*p* value
ENSG00000007541	PIGQ	Up	Up	5.78	5.48E‐05
ENSG00000086504	MRPL28	Up	Up	5.71	6.02E‐05
ENSG00000239920	AC104389	Up	Up	4.00	6.91E‐19
ENSG00000254473	AL354920	Up	Up	2.36	4.00E‐06
ENSG00000261150	EPPK1	Down	Down	−3.24	2.94E‐12
ENSG00000275395	FCGBP	Down	Down	−2.86	1.39E‐09
ENSG00000162804	SNED1	Down	Down	−2.53	1.16E‐06
ENSG00000049769	PPP1R3F	Down	Down	−2.42	4.44E‐06
ENSG00000075275	CELSR1	Down	Down	−2.14	1.86E‐03
ENSG00000207496	SNORA7A	Up	Down	−4.73	1.15E‐10
ENSG00000279339	AC100788	Up	Down	−3.80	1.69E‐05
ENSG00000286999	AL121581	Up	Down	−3.43	1.72E‐03
ENSG00000280213	UCKL1‐AS1	Up	Down	−3.32	6.41E‐05
ENSG00000229151	AC233976	Up	Down	−3.26	4.35E‐03

### 3.5 STRING interaction analysis and expression levels of KBD‐related genes

**FIGURE 5:**
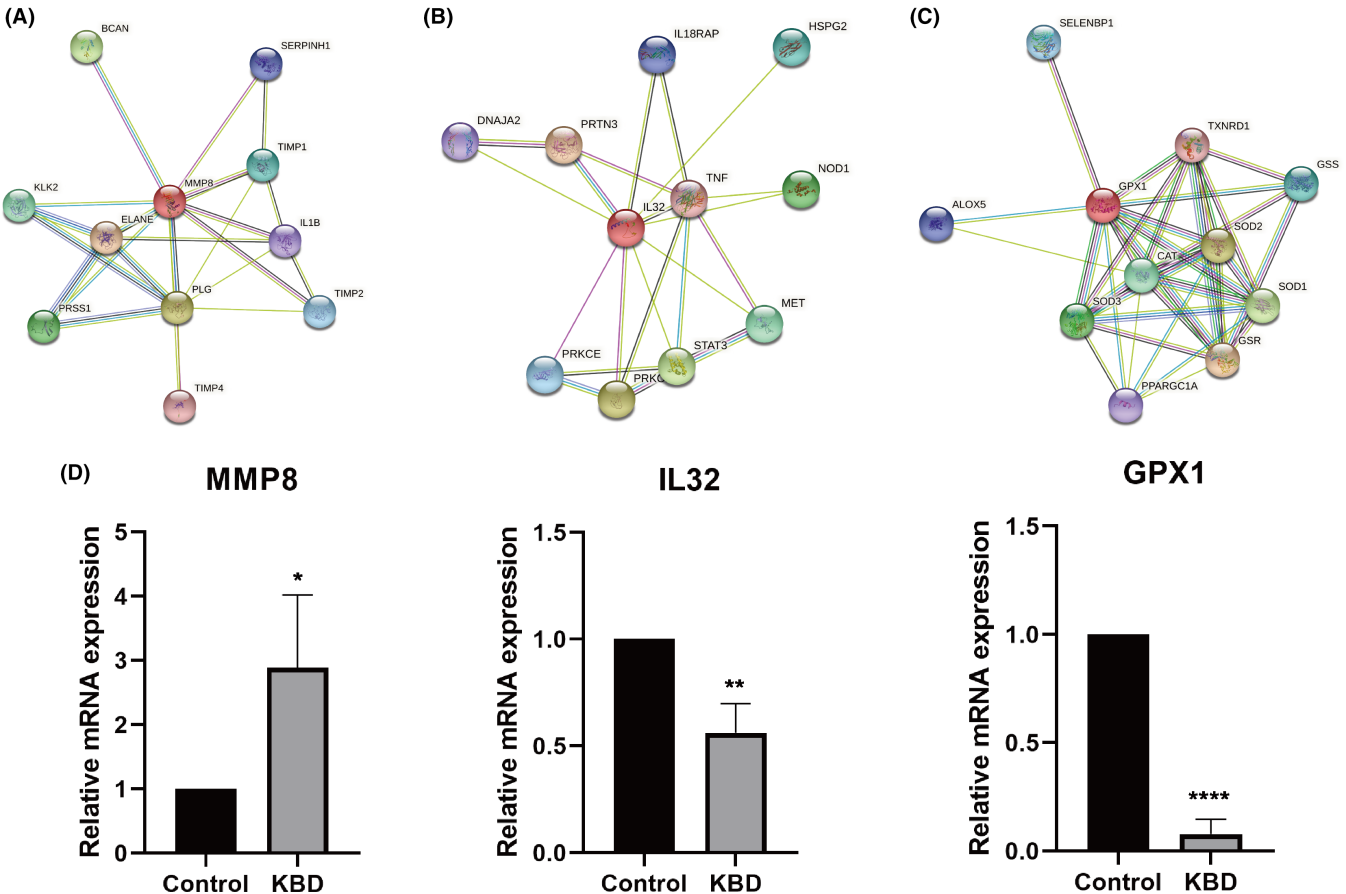
PPI network analysis and expression of m6A modification‐related factors in KBD. (A–C) PPI network of MMP8, IL32 and GPX1. All networks with evidence from experimental protein–protein interactions (PPI) (purple lines), text‐mining (bright green lines), and curated (turquoise blue lines) databases. (D) Quantitative analyses of MMP8, IL32 and GPX1 in peripheral blood. *p = 0.0448(MMP8), **p = 0.0051(IL32), ****p < 0.0001(GPX1).

Based on the RNA‐seq data, we screened three genes related to KBD. Including inflammatory factor MMP8, interleukin IL32 and selenoprotein GPX1.Their *p*‐values are 0.0187, 0.0291 and 0.0003 respectively. STRING interaction analysis respectively showed the roles of MMP8, IL32 and GPX1 in KBD (Figure 5A–C). The PPI network centered on MMP8 is strongly related to the cartilage damage‐related TIMP gene family and cytokine IL1β. IL32 is related to TNF, IL18 and the regulator of cytokines and growth factors STAT3. Within the differentially expressed gene list, MMP8, IL‐32 and GPX‐1 were selected to verify the expression using RT‐qPCR. The results of RT‐qPCR were similar with the RNA‐seq that the expression of MMP8 was significantly up‐regulated, while IL‐32 and GPX1 were significantly down‐regulated (Figure 5D).

## 4 DISCUSSION

KBD is an endemic bone and joint disease. Many studies have shown that epigenetic mechanism plays an important role in the pathogenesis of KBD.^21^, ^22^, ^23^, ^24^, ^25^, ^26^, ^27^ Our study focused on the m6A modification profile in KBD. In our study, using m6A MeRIP‐Seq and RNA‐seq, we depicted the transcriptome‐wide m6A methylome in KBD for the first time. The result found the distribution of m6A peaks and islands of m6A conserved motifs on different gene elements. From the sequencing results, the overall m6A modification level of KBD patients was higher than that of NCs. The distribution and expression of m6A peaks in chromosomes are unbalanced. The most differentially expressed methylated m6A peaks is distributed on chromosome 1. Among all 22 pairs of human autosomes, chromosome 1 contains the largest number of genes, accounting for more than 10% of all genes.^28^ Many diseases have been shown to be associated with gene mutations on chromosome 1, such as congenital heart disease,^29^ Multiple Myeloma,^30^ and cystic kidney disease.^31^ KBD patients may also have changes in gene expression on chromosome 1, resulting in changes in m6A modification levels.

Meanwhile, the result of GO analysis showed that the m6A DEGs are widely involved in KBD‐related pathways. One study showed that the apoptosis pathway, NF‐kappa B signalling pathway and the glutathione metabolism pathway were significantly enriched in KEGG pathway network,^32^ suggesting that these pathways may play key roles in KBD occurrence and development. The WNT/β‐catenin signalling pathway is suppressed in KBD, which induces chondrocyte apoptosis, leading to cartilage injury.^33^ TGF‐β is a pleiotropiccytokine that regulates joint homeostasis. There is noconsensus on the role of the TGF‐β/BMP signalling pathways on articular cartilage development and degradation.^34^ One aspect of apoptosis regulation is the PI3K/Akt signalling pathway, which plays an important role in the stimulation of apoptotic signals. PI3K and its downstream Akt kinase are important in cell survival and apoptosis. In particular, the apoptosis of chondrocytes is regulated by the PI3K/Akt signalling pathway, which is closely related to the occurrence and development of KBD.^35^ PRCAKA may be a gene that effect the development of KBD disease through Wnt signalling pathway. Compared to osteoarthritis animals before treatment, strong upregulation of PRKACA mRNA expression (approximately 3–5 fold change).^36^ THBS3 has significant change in the PI3K Akt signalling pathway. A study showed that THBS3 was identified as a target gene in the drug treatment of osteoarthritis and the expression of THBS3 was up‐regulated after treatment.^37^ IRS1 changed significantly in the autophagy pathway. A researcher found the IRS1 has been documented to modulate regulate synthesis of ECM proteins during the early development of chondrocytes.^38^ Further research on the relationship between m6A modification and RNA expression is the key content needed to focus on. We found that there were six hyper‐up genes (e.g. PIGQ and MRPL28), 23 hypo‐down genes (e.g. FCGBP and CELSR1), 307 hyper‐down genes (e.g. SNORA7A and AC100788). FCGBP and CELSR1 have been reported in bone disease research and the most of others are recently discovered new transcripts or genes, so there are few related studies. FCGBP, namely Fc gamma binding protein, was identified as being associated with osteosarcoma metastasis. The researchers also performed GO and KEGG analysis and found that FCGBP play a role in cancer‐related biological processes, such as apoptosis and biosynthetic processes.^39^ CELSR1 mutation can reduce trabecular bone mass and distal tibial cortical thickness in mice. Studies have shown that PCP signalling disruption caused by Celsr1 (Celsr1 Crsh/+) mutations significantly reduced trabecular bone mass and distal tibial cortical thickness.^40^ These reports suggest that subsequent studies on the aetiology of KBD should focus on the impact of these genes on the onset and course of KBD.

In the final stage of this research, we selected MMP8, IL‐32, GPX1 that were significantly dysregulated to verify using RT‐qPCR. The relative mRNA expressions of MMP8, IL32 and GPX1 were consistent with the sequencing results. Further we explored the biological functions of these three genes and construct the PPI network. MMP8 as one member of the MMP family, participates in the cartilage matrix metabolism, besides it is closely related to cartilage inflammation‐related cytokine IL1β and TIMP gene family. IL32 is related to TNF, IL18 and the regulator of cytokines and growth factors STAT3. STRING analysis provides us with an exploration direction to study the interaction between these genes. MMPs are a family of zinc‐containing endopeptidases with overlapping specificities that are involved in the degradation of different extracellular matrices (ECM) in KBD cartilage.^41^ One study showed a significant difference in the phenotypic expression of MMP‐13 in KBD and control chondrocytes, suggesting degenerative and hypertrophic changes in chondrocytes of KBD articular cartilage.^42^, ^43^ However, there is no previous study about MMP8 in KBD. IL‐32 has demonstrated proinflammatory cytokine properties in bone disease.^44^ Although IL‐32 also has not been studied in KBD, its family of IL‐6 and IL‐1β have been shown to be upregulated to varying degrees.^45^ T‐2 toxin under selenium‐deficient nutrition induces elevated levels of IL‐6 and IL‐1β in serum and cartilage, which may explain the pathological mechanism of cartilage injury in KBD.^46^ Among the 25 selenoproteins found in mammals, the transcript levels of GPX1, GPX4 and Sep15 were decreased in KBD patients, suggesting that selenoprotein gene polymorphisms are closely related to the susceptibility of KBD to low selenium environments.^47^ This study has several limitations. The peripheral blood samples we collected were limited, more blood and chondrocyte samples are needed to further study the role of m6A in KBD.

In conclusion, our study provided the transcriptome‐wide RNA m6A methylation profiles in KBD. We analysed differences in m6A modification in KBD patients relative to NCs and demonstrated a strong association between m6A modifications and KBD progression. The study paves the clues for future researches aimed at exploring the pathogenesis of KBD.

## AUTHOR CONTRIBUTIONS

**Qian Zhang:** Writing – original draft (equal). **Xiaodong Yang:** Data curation (equal); formal analysis (equal). **Xingxing Deng:** Methodology (equal); visualization (equal). **Hui Niu:** Validation (equal). **Yijun Zhao:** Investigation (equal). **Jinfeng Wen:** Visualization (equal). **Sen Wang:** Resources (equal). **Huan Liu:** Resources (equal). **Xiong Guo:** Project administration (equal). **Cuiyan Wu:** Funding acquisition (equal); supervision (equal).

## FUNDING INFORMATION

This study was supported by the National Natural Scientific Foundation of China (82073495).

## CONFLICT OF INTEREST STATEMENT

The authors declare that they have no competing interests.

## Supporting information


Table S1.
Table S2.
Table S3.

## Data Availability

The data underlying this article will be shared on reasonable request to the corresponding author.
