# Supplementary material for: Transcriptome‐wide RNA m6A methylation profiles in an endemic osteoarthropathy, Kashin‐Beck disease
Source: J Cell Mol Med. 2024 Oct 20;28(20):e70047. doi: 10.1111/jcmm.70047 (PMC11491295; doi:10.1111/jcmm.70047)
Supplement: Supplementary file 1 — Table S1. Table S2. Table S3. [file JCMM-28-e70047-s001.docx]

Supplementary table 1. The specific information of subjects.

| Subjects | Sex | Age (years) | Disease degree |
| --- | --- | --- | --- |
| KBD-1 | Female | 63 | Ⅱ |
| KBD-2 | Male | 59 | Ⅱ |
| KBD-3 | Male | 65 | Ⅱ |
| Normal-1 | Male | 58 | / |
| Normal-2 | Female | 65 | / |
| Normal-3 | Male | 62 | / |

Supplementary table 2. m6A regulators differently expressed in KBD.

| Gene ID | Gene name | Log2FC | Regulation | P value |
| --- | --- | --- | --- | --- |
| ENSG00000145388 | METTL14 | 0.56 | up | 9.33E-04 |
| ENSG00000083896 | YTHDC1 | -1.01 | down | 2.00E-19 |
| ENSG00000164944 | VIRMA | 1.27 | up | 1.32E-06 |
| ENSG00000123200 | ZC3H13 | -1.50 | down | 2.63E-07 |
| ENSG00000091542 | ALKBH5 | 1.62 | up | 3.47E-08 |

Supplementary table 3. Quantitative PCR primers.

| Gene | Forward (5' to 3') | Reverse (3' to 5') |
| --- | --- | --- |
| VIRMA | AAGTGCCCCTGTTTTCGATAG | ACCAGACCATCAGTATTCACCT |
| ALKBH5 | CGGAAGTACCAGGAGGACTCAGAC | GGATGCCGCTCTTCACCTTGC |
| YTHDC1 | GGAAAAGGGATCCTGAAAGG | CTGCCAGTCTCATGGTCAGA |
| ZC3H13 | TCTGATAGCACATCCCGAAGA | CAGCCAGTTACGGCACTGT |
| METTL14 | AGAAACTTGCAGGGCTTCCT | TCTTCTTCATATGGCAAATTTTCTT |
| MMP8 | GAGGACAGAAAGAAAGCCAGGAG | AACTTTTCCAGGTAGTCCTGAA |
| IL32 | ACGACTTCAAAGAGGGCTAC | TCCTCAACATCCGGGACAGG |
| GPX1 | TTCCCGTGCAACCAGTTTG | TTCACCTCGCACTTCTCGAA |
| GAPDH | GAAGGTGAAGGTCGGAGTC | GAAGATGGTGATGGGATTTC |
